# Effects of Atrazine, Metolachlor, Carbaryl and Chlorothalonil on Benthic Microbes and Their Nutrient Dynamics

**DOI:** 10.1371/journal.pone.0109190

**Published:** 2014-10-02

**Authors:** Daniel Elias, Melody J. Bernot

**Affiliations:** Ball State University, Department of Biology, Muncie, Indiana, United States of America; Texas Tech University, United States of America

## Abstract

Atrazine, metolachlor, carbaryl, and chlorothalonil are detected in streams throughout the U.S. at concentrations that may have adverse effects on benthic microbes. Sediment samples were exposed to these pesticides to quantify responses of ammonium, nitrate, and phosphate uptake by the benthic microbial community. Control uptake rates of sediments had net remineralization of nitrate (−1.58 NO_3_ µg gdm^−1^ h^−1^), and net assimilation of phosphate (1.34 PO_4_ µg gdm^−1^ h^−1^) and ammonium (0.03 NH_4_ µg gdm^−1^ h^−1^). Metolachlor decreased ammonium and phosphate uptake. Chlorothalonil decreased nitrate remineralization and phosphate uptake. Nitrate, ammonium, and phosphate uptake rates are more pronounced in the presence of these pesticides due to microbial adaptations to toxicants. Our interpretation of pesticide availability based on their water/solid affinities supports no effects for atrazine and carbaryl, decreasing nitrate remineralization, and phosphate assimilation in response to chlorothalonil. Further, decreased ammonium and phosphate uptake in response to metolachlor is likely due to affinity. Because atrazine target autotrophs, and carbaryl synaptic activity, effects on benthic microbes were not hypothesized, consistent with results. Metolachlor and chlorothalonil (non-specific modes of action) had significant effects on sediment microbial nutrient dynamics. Thus, pesticides with a higher affinity to sediments and/or broad modes of action are likely to affect sediment microbes' nutrient dynamics than pesticides dissolved in water or specific modes of action. Predicted nutrient uptake rates were calculated at mean and peak concentrations of metolachlor and chlorothalonil in freshwaters using polynomial equations generated in this experiment. We concluded that in natural ecosystems, peak chlorothalonil and metolachlor concentrations could affect phosphate and ammonium by decreasing net assimilation, and nitrate uptake rates by decreasing remineralization, relative to mean concentrations of metolachlor and chlorothalonil. Our regression equations can complement models of nitrogen and phosphorus availability in streams to predict potential changes in nutrient dynamics in response to pesticides in freshwaters.

## Introduction

Agricultural activities, such as crop protection via pesticides, are increasing in response to global human population growth (projected to reach 9 billion by 2050; [1]). In the last decade, U.S. pesticide sales have increased ∼10% with 80% of these pesticides used for agricultural activities [1]. The continued growth of the human population, coupled with the need for more efficient agricultural practices, will undoubtedly yield future increases in the occurrence of pesticides in freshwaters. Further, despite decades of research on agricultural pesticides, recent calls to action have highlighted the need to fill critical knowledge gaps in our understanding of how pesticides may adversely affect aquatic ecosystems [2], [3].

Once in the aquatic ecosystem, pesticides may have adverse effects on organisms ranging from direct toxicity to indirect effects such as changes in growth or behavior [4]. At higher concentrations, such as those following spring runoff, direct mortality results across diverse organisms including tadpoles [4], bluegill [5], and benthic organisms (e.g., amphipods and chironomids, [6]). However, at lower concentrations, sub-lethal effects can result in altered respiration rates [7], organismal growth [4] and fecundity [8]. In streams, benthic microbes are an important component of aquatic ecosystems and are integral in nutrient and energy dynamics [9]. For example, nitrate, ammonium and phosphorus are cycled by benthic microbes through assimilation and remineralization [10]. These processes are influenced by N concentration in freshwaters [11], [12]. At saturated conditions, such as those in agricultural streams with high input of N from fertilizer runoff, more N is available, due to microbial uptake saturation (i.e. biota have reach their N demand; [13]) or an increase in heterotrophic mineralization [11]. Further, at increasing N concentrations, PO_4_ often becomes a secondary limiting nutrient [12].

In the Midwestern U.S., two herbicides (atrazine and metolachlor), one insecticide (carbaryl) and one fungicide (chlorothalonil) have both high usage rates and prevalence in receiving waters [14]–[17]. Atrazine is a triazine herbicide used predominantly in corn production for control of broadleaf and grassy weeds [18] with a half-life in water at pH seven of 86 days [19]. Metolachlor is a chloroacetanilide herbicide that inhibits mitosis and cell division [19]. Metolachlor is stable in water at pH seven [19]. Atrazine and metolachlor were detected in U.S. freshwaters at peak concentrations of 201 µg/L and 77.6 µg/L (Table 1). Carbaryl is a carbamate family insecticide that inhibits the enzyme acetylcholinesterase [19], with a half-life in water of 12 days at pH seven [19]. Chlorothalonil is a fungicide used in U.S. agriculture; it is stable in water at pH seven [19]. Its mode of action is by binding to glutathione and negatively affecting cellular respiration [7]. Carbaryl and chlorothalonil were detected in U.S. freshwaters at peak concentrations of 4.8 µg/L and 0.3 µg/L (Table 1).

**Table 1:** Detection frequency and concentrations of atrazine, metolachlor, carbaryl, and chlorothalonil in U.S. freshwaters.

Compound	Detection frequency (%)	Mean concentration (µg/L)	Maximum concentration (µg/L)	References
*Atrazine (Herbicide)*	78.1	2.4	201	[15]–[17]
*Metolachlor (Herbicide)*	71.1	1.2	77.6	[15]–[17]
*Carbaryl (Insecticide)*	18.1	0.013	4.78	[15]–[17]
*Chlorothalonil (Fungicide)*	0.033	<0.07	0.29 (290*)	[15]–[17]

Detection frequency and concentrations of atrazine, metolachlor, carbaryl, and chlorothalonil in U.S. freshwaters. Detection frequency was estimated throughout the U.S. across 50 basins (33 agricultural, 10 urban and 7 mixed); mean and maximum concentrations correspond to 83 agricultural streams. Annual mean detection frequencies for each compound at each site provide the proportion of water samples that have detectable levels of pesticides for a year period. *Chlorothalonil was detected at concentrations of 290 µg/L in run-off near golf courses.

These pesticides are detected in freshwater ecosystems at concentrations that adversely affect biota and human health (Table 2). However, research has focused primarily on the impacts of agricultural pesticides on fish and invertebrates; little is known about how exposure to pesticides may directly influence benthic nutrient dynamics and overall ecosystem function [20]. For example, ecotoxicology studies addressing the effects of pesticides focus primarily on non-benthic vertebrates (e.g., bluegill, [5]), and benthic invertebrates (e.g., amphipods and chironomids, [6]) with few studies conducted on sediment microbial dynamics [21]. Benthic microbial communities influence nutrient cycling (e.g. uptake, remineralization) by affecting fluxes as consumers or sources. Thus, benthic microbial communities are an important component of the freshwater ecosystem [22]. These nutrient dynamics are affected by the presence of pesticides [23]. Specifically, pesticides can reduce microbial activity that contributes to nutrient cycling (e.g. *Volvox* spp., *Botryococcus* spp., *Synedra* spp.) [24], and change species composition by favoring microbes with enhanced pesticide degradation capacities. Also, pesticides can become nutrient sources by providing carbon, nitrogen or phosphorus to some microorganisms [25], and alter nitrogen and/or phosphorus cycles [26]. Thus, there is a need to understand the direct effect of pesticides on sediment nutrient dynamics, and how these changes can affect whole-ecosystem pools and fluxes of nutrients [27].

**Table 2:** Toxicity and Octanol-water partition coefficient of atrazine, metolachlor, carbaryl and chlorothalonil to daphnids, green algae, and humans.

Compound	Daphnids	Green algae	Humans	Octanol-Water partition coefficient
	(mg/L)	(mg/L)	(mg/kg/bw/d)	
	NOEC	NOEC	ADI	Log K_ow_
*Atrazine*	0.25	0.1	0.02	2.7
*Metolachlor*	0.7	57.1*	0.1	3.4
*Carbaryl*	0.25	-	0.0075	2.4
*Chlorothalonil*	0.009	0.033	0.015	2.9

Toxicity of atrazine, metolachlor, carbaryl and chlorothalonil in mg/L to daphnids and green algae, and mg/kg of body weight (bw) per day (d) to humans. No observed effect concentrations (NOEC) for daphnids and green algae were calculated by chronic tests of 21 days and 96 hours, respectively. Acceptable daily intake (ADI).* Half maximal effective concentration (EC50) of metolachlor on growth after 72 hours (19).

We measured the effects of atrazine, metolachlor, carbaryl and chlorothalonil on benthic microbial nutrient dynamics by quantifying net assimilation and remineralization rates of ammonium, nitrate and phosphate in laboratory mesocosms. We hypothesized that pesticides with a broad mode of action and higher affinity to organic matter such as chlorothalonil (disruption of cellular respiration, log K_ow_: 2.9) and metolachlor (inhibitor of mitosis and cellular division, log K_ow_: 3.4) would decrease microbial nitrate and phosphate uptake rates. In contrast, pesticides with more specific modes of action and higher affinity to the aqueous phase (atrazine: blocks photosynthesis, log K_ow_: 2.7 and carbaryl: inhibitor of synaptic activity, log K_ow_: 2.4) were predicted to have no effect on nutrient cycling. Further, natural resources managers and stakeholders would be able to make general predictions of agricultural pesticides effects on the microbial community based on the mode of action and water/solid affinities of these pesticides.

## Materials and Methods

### Experimental mesocosms

Sediment and water collection was conducted in May 2012 at Ball State University field station property of Jakes Creek - Cooper farm/Skinner field (40.234493, −85.45235) and approval for these experiments was received following the appropriate procedures. This sample collection did not involve endangered or protected species. Jakes Creek is a 3rd order agriculturally-influenced stream with adjacent row crops (i.e. corn and soybean) in Muncie, Indiana within the Upper White River Watershed (UWRW). During the sampling time, Jakes Creek water temperature was 17°C, pH 7.92, depth 5 cm (at sampling location), and discharge was 37 L/s. -While stream samples were not collected for pesticides analysis at the time of this experiment; stream water and sediment samples were collected one week prior to this study at the same site that shows atrazine and metolachlor concentrations were below detection limits. Filtered water samples (ten 1000 mL and one 200 mL) were collected from the stream thalweg using a 60 mm syringe and subsequently filtered (Whatman© glass fiber filter; 0.7 µm nominal pore size) into acid-washed Nalgene© bottles. The 200 mL Nalgene plastic bottle was used to determine initial concentrations of nitrate, ammonium, and phosphate. A composite sediment sample (∼2000 cm^3^) was randomly collected from the top 5 cm of the stream benthos and placed into three Nalgene plastic bottles. Sediment samples were transported on ice and subsequently combined and homogenized using a USGS no. 5 sieve in the laboratory. Homogenized sediment (20 cm^3^) and 60 ml filtered stream water were placed into each of 160 laboratory mesocosms (Fisherbrand sterile urine cup, 120 ml).

Stock solutions were prepared for atrazine (Atrazine 4L, 42.2% purity, Loveland, CO, US), metolachlor (Me-too-lachlor II, 84.4% purity, Drexel Chemical Company, TN, US), carbaryl (Sevin XLR Plus, 44.1% purity, Bayer, NC, US), and chlorothalonil (Bravo, 54% purity, Syngenta, NC, US) to achieve final stock concentrations of 10,000 µg/L for atrazine and metolachlor, 5,000 µg/L for carbaryl, and 8,000 µg/L for chlorothalonil. Aliquots from each stock solution were added to mesocosms to reach ten target treatment concentrations for each pesticide with four replicates for each treatment.

Mean and peak atrazine (2 µg/L, 201 µg/L) and metolachlor (1 µg/L, 78 µg/L) are detected at concentrations ∼2–3 orders of magnitude higher than carbaryl (0.01 µg/L, 5 µg/L) or chlorothalonil (0.07 µg/L, 0.3 µg/L) throughout the U.S. (14–17). Thus, the treatment concentrations used were selected to include these environmentally relevant concentrations and appropriately represent pesticide occurrence in streams. Treatment solutions used in this study ranged from 0 µg/L (control) to the maximum concentrations detected in U.S. freshwaters for atrazine (200 µg/L), metolachlor (80 µg/L), carbaryl (4 µg/L) and chlorothalonil (0.5 µg/L) (Table 2).

Water from each mesocosm was removed after 24 h using a 10 mL syringe, subsequently filtered as above and placed into vials (two analytical replicates ∼5 ml) for analysis of nitrate and phosphate via ion chromatograph (DIONEX, ICS-3000). The colorimetric phenol-hypochlorite technique [28], [29] was used to quantify ammonium concentrations. Initial concentrations (background) of nitrate, ammonium, and phosphate were also analyzed following the analytical methods above. Sediment dry mass in each mesocosm was quantified using an analytical balance (OHAUS, Adventurer SL AS64).

### Data analysis

Nutrient uptake rates were calculated for phosphate, ammonium and nitrate as changes in concentration over time (24 h) per g of dry mass (sediment) in response to treatments as (22):
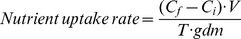

*Where: Cf*  =  Final concentration (mg/L); *Ci*  =  Initial concentration (mg/L); *V*  =  Volume (L) in the jar*T*  =  time (h); *gdm*  =  g dry mass (g). Negative nutrient uptake rates indicated net remineralization of nutrients and positive nutrient uptake rates indicated net assimilation of nutrients (Table 3). Uptake rates were divided by the average of the control treatment for each pesticide (N = 16) to assess the effects of different treatments for a particular pesticides. Thus, in this study, a response ratio relative to controls was used as the response variable. Further, data were log-transformed to meet normality assumptions for statistical analyses. SigmaPlot^©^ 12.0 software was used for linear and nonlinear regression analyses of response to pesticide concentration. The Akaike Information criterion (AIC) was used to select the best fit model among the different polynomial candidate models. Further, to develop predictive models of microbial response to pesticides in agricultural waters mean and peak concentrations of metolachlor and chlorothalonil throughout U.S. freshwaters (Table 1) were used to calculate nitrogen and phosphorus uptake rates.

**Table 3:** Uptake rates for nitrate, phosphate, and ammonium in response to pesticides exposure.

Pesticide	Nitrate (µg gdm^−1^ h^−1^)	Phosphate (µg gdm^−1^ h^−1^)	Ammonium (µg gdm^−1^ h^−1^)
*Atrazine*	−25.88 (−45.2–4.30)	126.93 (83.1–169)	0.36 (0.23–0.45)
*Metolachlor*	−21.34 (−40.9–2.10)	169.54 (120–214)	0.39 (0.03–0.53)
*Carbaryl*	−25.13 (−56.6–8.80)	192.11 (37.9–260)	0.31 (0.13–0.53)
*Chlorothalonil*	−56.54 (−83.1–−3.7))	233.67 (39.8–601)	0.28 (0.13–0.47)
*Mean uptake rate*	**−32.22**	**180.56**	**0.34**
*Control*	−1.58 (−1.62–−1.49)	1.34 (7.03E-05–4.4)	0.03 (0.01–0.04)

Uptake rates for nitrate, phosphate, and ammonium (µg gdm^−1^ h^−1^) across pesticide and control (no pesticide) treatments. Range noted in parentheses. Mean uptake rate was calculated for each nutrient across pesticides.

## Results

Nitrate, phosphate, and ammonium uptake rates varied less than one order of magnitude across pesticide treatments (Table 3). Phosphate uptake rates were three orders of magnitude greater than ammonium uptake rates across treatments, though both phosphate (mean  = 180.56 µg gdm^−1^ h^−1^) and ammonium (mean  = 0.34 µg gdm^−1^ h^−1^) uptake rates were net assimilative (Table 3). In contrast, we observed net remineralization of nitrate (mean  = −32.22 µg gdm^−1^ h^−1^) across pesticides (Table 3). Overall, these nutrient dynamics are expected in nitrogen-saturated agricultural ecosystems (i.e., microbial nitrate assimilation is saturated and PO_4_ becomes a limiting nutrient).

### Nitrate dynamics

**Figure 1:**
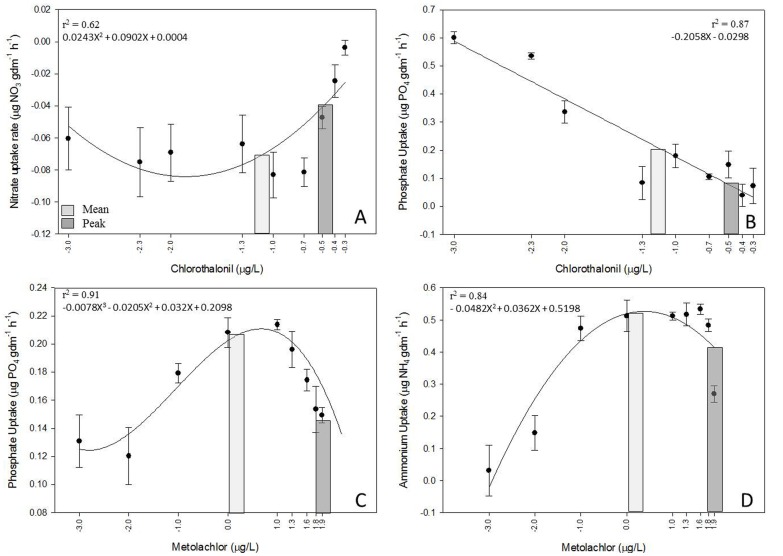
Nutrient uptake rates (mean +/− SE) response to pesticide concentrations after 24 h incubation (4 replicates, 10 treatments, N = 40). X-axis is in a Log10 scale. A: Nitrate uptake in response to chlorothalonil concentrations. B: Phosphate uptake rate response to chlorothalonil concentrations. C: Phosphate uptake rate response to metolachlor concentrations. D: Ammonium uptake rate response to metolachlor concentrations. Columns represent predicted uptake rates for each nutrient calculated at mean and peak concentrations of metolachlor and chlorothalonil measure in U.S. freshwaters (Table 2).

Control nitrate uptake rate was ∼20x higher on average than nitrate uptake influenced by pesticides (i.e., increasing remineralization in presence of pesticides). Nitrate uptake rates in response to atrazine, metolachlor, and carbaryl treatments exposure ranged from net assimilation (consumption/removal) to remineralization (source/addition) in the water column of our mesocosms (Table 3). In contrast, increasing concentrations of chlorothalonil yielded increasing nitrate remineralization (p = 0.02, r^2^ = 0.83, Fig. 1A). No other pesticides had significant effects on nitrate uptake rates (p>0.05).

### Phosphate dynamics

Phosphate uptake rates in response to pesticides exposure was ∼100x higher than control phosphate uptake rates. Thus, phosphate assimilation increased in the presence of atrazine, metolachlor, carbaryl, and chlorothalonil relative to the control (Table 3). Further, phosphate uptake was negatively related to increasing chlorothalonil concentration (p<0.001, r^2^ = 0.87, Fig. 1B). Similarly, the metolachlor effect on phosphate uptake rate (p = 0.005, r^2^ = 0.91, Fig. 1C) followed a cubic relationship with increasing phosphate uptake at lower concentrations (0 to 10 µg/L), and decreasing rates at higher concentrations (10 to 80 µg/L). No other pesticides resulted in significant effects on phosphate uptake rates (p>0.05).

### Ammonium dynamics

Ammonium uptake rates in response to pesticides exposure was ∼10x higher than control ammonium uptake rate. Thus ammonium assimilation increased in the presence of atrazine, metolachlor, carbaryl, and chlorothalonil relative to the control (Table 3). The ammonium uptake rate varied in response to metolachlor treatments (p = 0.023, r^2^ = 0.83, Fig. 1D), increasing with lower concentrations of metolachlor (0 to 10 µg/L), followed by a decline at higher concentrations (10 to 80 µg/L). No other pesticides had significant effects on ammonium uptake rates (p>0.05).

### Predicting nutrient response to pesticides

Changes in stream ecosystem nitrogen and phosphorus uptake rates were modeled across metolachlor and chlorothalonil concentrations detected throughout the U.S. (Fig. 1). These changes were generated with polynomial regressions from this experiment (p<0.05).

The predicted ammonium uptake (0.52 NH_4_ µg gdm^−1^ h^−1^) in response to mean metolachlor concentrations was ∼20% higher than ammonium uptake (0.42 NH_4_ µg gdm^−1^ h^−1^) at peak metolachlor concentrations. At mean concentrations of metolachlor, the predicted phosphate uptake (212.2 PO_4_ µg gdm^−1^ h^−1^) was ∼40% lower than phosphate uptake (372.41 PO_4_ µg gdm^−1^ h^−1^) at peak metolachlor concentrations. At mean and peak chlorothalonil concentrations, there is net remineralization of nitrate (−71.99 NO_3_ µg gdm^−1^ h^−1^ and −41.87 NO_3_ µg gdm^−1^ h^−1^, respectively). Further, there was net assimilation of phosphate in response to mean and peak concentrations of chlorothalonil (207.88 and 77.81 PO_4_ µg gdm^−1^ h^−1^, respectively).

Overall, there was decreased ammonium assimilation and remineralization of nitrate in the presence of peak concentrations of metolachlor and chlorothalonil (Fig. 1). Phosphate assimilation increased at peak concentrations of metolachlor and decreased at peak concentrations of chlorothalonil (Fig. 1). Peak chlorothalonil is predicted to have the greatest effect on phosphate and nitrate uptake rates, by decreasing net assimilation and remineralization, respectively, over 50% relative to mean concentrations of these pesticides.

## Discussion

Research focuses primarily on the impacts of agricultural pesticides on fish and invertebrates [5], [6]; however, little is known about effects on microbial communities [20]. Our ecotoxicological research showed that benthic microbes' nutrient uptake rate response is likely a function of pesticide chemical characteristics, and how these changes can affect nutrient dynamics, due to nutrient availability.

Variation in nutrient uptake rates is likely a result of differences in baseline nutrient concentrations and the biotic community. For example, control ammonium uptake rates in this study (0.026 µg NH_4_ gdm^−1^ h^−1^) were ∼3x lower than control ammonium uptake rates in Bunch and Bernot [30]. These results suggest dissimilar nutrient demand due to unique biotic communities and nutrient conditions across sampling sites and temporal variability.

In our study, mean ammonium uptake rate was ∼1.5x higher than ammonium uptake under enriched nitrate conditions and ∼2.5x lower under enriched ammonium conditions reported in Bunch and Bernot [30]. Thus, ammonium uptake is higher in the presence of pesticides than under enriched nitrate conditions and lower under enriched ammonium conditions, due to microbial adaptations to nutrient availability and metabolic responses in presence of pesticides. Our nitrate uptake results were similar to yields with net remineralization under enriched ammonium conditions reported in Bunch and Bernot [30], though remineralization was 10 times lower than rates measured in response to pesticides in our study.

The effects of metolachlor and chlorothalonil on nutrient uptake rates suggest a unique biotic community at this site, represented by mostly heterotrophic benthic microbes. Our results suggest a toxic effect of metolachlor and chlorothalonil on the benthic microbial community that is reflected by the increased nitrate remineralization, and reduced assimilation of ammonium and phosphate. This increase in remineralization rates could be an outcome of cellular lysis or a stress mechanism [31]. Further, a biotic community characterized by autotrophs [22] had ammonium and nitrate uptake rates three orders of magnitude higher than benthic uptake in our study; possibly, under these study conditions, primary producers have a higher assimilation rate than heterotrophic benthic microbes in response to the available forms of nitrogen [22]. Further, control phosphate uptake rates in our study were ∼5x lower than rates previously measured with phosphorus enrichment (0.1–2 mg/L; 34). Thus, phosphate is likely a limiting nutrient in our system. However, in presence of pesticides, phosphate uptake was two orders of magnitude higher than the rates measured under phosphorus limiting conditions [32] possibly, in addition to available phosphorus in the water column, microbes were potentially degrading pesticides as a source of phosphorus [33].

Nutrient dynamics in the presence of pesticides are dependent on the physicochemical characteristics (e.g. sorption kinetics, modes of action) of each pesticide. Sorption kinetics of pesticides and their corresponding index (Octanol-water partition coefficient, K_ow_) determine the affinity of organic contaminants to either the water column or sediments [34]. Atrazine and carbaryl have a higher affinity to the aqueous phase (Log K_ow_: 2.7 and 2.4, respectively), relative to metolachlor and chlorothalonil (Log K_ow_: 3.4 and 2.9, respectively). Thus, atrazine and carbaryl are likely more prevalent in the water column and less available to the sediment microbial communities, with minimal effect on benthic microbial activity. In contrast, metolachlor and chlorothalonil have a higher affinity to solids and higher prevalence in sediment, potentially affecting benthic microbial activity. Our interpretations of pesticide availability based on their water/solid affinities supports our results of no effects for atrazine and carbaryl (Log K_ow_<2.7, p>0.05) within the tested range, decreasing nitrate remineralization and decreasing phosphate uptake in response to chlorothalonil, and decreasing ammonium and phosphate uptake in response to metolachlor (Log K_ow_>2.9, p<0.05).

Nutrient dynamics are also affected by the pesticide mode of action. Atrazine and carbaryl have specific modes of action; atrazine blocks photosynthesis, and carbaryl inhibits the activity of acetylcholinesterase, an enzyme of insects, fish, mammals [35]. Thus, the specificity of atrazine and carbaryl, and a sediment microbial community dominated by heterotrophs with no synaptic activity [36] may explain the lack of significant effects of these pesticides on benthic microbes within the tested range (Table 3). In contrast, metolachlor and chlorothalonil are broad spectrum pesticides [19]. In our study, ammonium and phosphate uptake rates decreased with increasing metolachlor concentrations. These decreasing ammonium and phosphate uptake rates could be due to metolachlor inhibition of mitosis and cell division. Similarly, phosphate and nitrate uptake rates were affected by chlorothalonil; possibly this fungicide affects benthic microbes by disrupting cellular respiration. Thus, pesticides with non-specific modes of action (e.g. metolachlor, chlorothalonil) are more likely to have a significant effect on nutrient dynamics of sediment microbes, consistent with our study results.

Metolachlor and chlorothalonil not only affect nutrient dynamics of the sediment microbial community; they can also affect other processes. In our study, at increasing concentrations of chlorothalonil, there is a decrease in phosphate uptake, thus there is more phosphate available for organismal consumption. In agricultural streams, where phosphorus is the limiting nutrient [37], increasing availability of phosphate can lead to algal blooms, eutrophication, hypoxia, loss of biodiversity (e.g. fish kills), and loss of aesthetic value of these habitats [38]. In contrast, in the presence of peak concentrations of metolachlor, there is an increase in phosphate assimilation, which could mitigate the excess phosphate in streams [39]. Similarly, there is an increase of ammonium availability due to inhibited uptake rates in response to metolachlor, which in turn could potentially increase biological activity of pesticide resistant microbes [21], [25], [26], [33]. Nitrate remineralization also decreases in response to chlorothalonil, reducing nitrate availability, and potentially further mitigating excess nitrogen in these habitats [40].

Mesocosms have a critical role in understanding the mechanisms driving ecological processes [42], [43] and provide a bridge between smaller, better controlled experiments and the larger freshwater ecosystems [44]. For example, [43] revealed that the effects of nutrients on primary producers are similar in artificial habitats across five orders of magnitude in size. Also, [44] mentioned that mesocosms help disentangle direct from indirect effects over scale. Thus, our conclusions try to bridge what we observed in the laboratory level and what could potentially occur at an agricultural influenced stream level.

Pesticide occurrence and concentrations in streams are dictated primarily by land-use. Streams receiving run-off from agricultural landscapes frequently have the highest concentrations of pesticides, compared to forested, mixed-use and urban lands [41]. These high concentrations occur as pulses that are coupled by the seasonality of agricultural practices [16]. Peak concentrations of metolachlor and chlorothalonil are detected from April to August [16]. Thus, the effects of these pesticides on nutrient dynamics are highest during this critical time. At peak metolachlor and chlorothalonil concentrations there is a decrease of predicted ammonium assimilation and nitrate remineralization. Further, at peak concentrations of metolachlor there is increased phosphate assimilation and at peak concentrations of chlorothalonil there is decreased phosphate assimilation (Fig. 1).

Our findings demonstrate individual effects of these pesticides on sediment nutrient dynamics that are likely driven by a pesticides' mode of action and water/sediment affinities. More studies are required to understand the net effect on ecosystems and to address their synergistic or antagonistic effects as mixtures. The regression equations we generated can complement models of nitrogen and phosphorus availability in streams to predict the potential changes in nutrient dynamics in response to increasing presence of pesticides in lotic ecosystems.
